# The Melbourne Assessment of Schizotypy in Kids: A Useful Measure of Childhood Schizotypal Personality Disorder

**DOI:** 10.1155/2015/635732

**Published:** 2015-01-06

**Authors:** Harvey P. Jones, Renee R. Testa, Nola Ross, Marc L. Seal, Christos Pantelis, Bruce Tonge

**Affiliations:** ^1^School of Psychology and Psychiatry, Monash University, Clayton, VIC 3800, Australia; ^2^Department of Psychiatry, Melbourne Neuropsychiatry Centre, University of Melbourne, Carlton South, VIC 3053, Australia; ^3^Child and Adolescent Neuropsychology Group, East Melbourne, VIC 3002, Australia; ^4^Murdoch Children's Research Institute, Royal Children's Hospital, Parkville, VIC 3052, Australia; ^5^Centre for Developmental Psychiatry and Psychology, School of Psychology and Psychiatry, Monash University, VIC 3800, Australia

## Abstract

Despite being identified as a high risk cohort for psychosis, there has been relatively little research on the clinical presentation and assessment of Schizotypal Personality Disorder (SPD) in childhood. The current study aimed to develop a measure of childhood SPD (Melbourne Assessment of Schizotypy in Kids (MASK)) and assess discriminant validity against another neurodevelopmental disorder, autism spectrum disorder (ASD). Sixty-eight children aged between 5 and 12 (21 SPD, 15 ASD, and 32 typically developing) and their parents were administered the MASK. The MASK is a 57-item semistructured interview that obtains information from the child, their parents, and the clinician. The results showed high internal consistency for the MASK and higher scores in the SPD group. A factor analysis revealed two MASK factors: social/pragmatic symptoms and positive schizotypal symptoms. Both factors were associated with SPD, while only the social/pragmatic factor was associated with ASD. Within the two clinical groups, a receiver operating characteristic curve showed that the MASK (cut-off score: 132 out of 228) was a good indicator of SPD diagnosis. These preliminary MASK findings were reliable and consistent and suggest that childhood SPD is characterised by complex symptomology distinguishable from ASD.

## 1. Introduction

Recent interest in the neurodevelopmental trajectories of schizophrenia spectrum disorders (SSDs) has led to an increased focus on the early developmental period [1, 2]. This research has identified neurocognitive and behavioral markers including developmental language delay [3], motor dysfunction [4], poor academic performance [5], and social difficulties [3, 6], which indicate risk for the later development of psychiatric disorders [7]. Child and adolescent patient groups that present with this cluster of deficits, as well as additional risk markers for the development of psychosis, have been identified in the community [8, 9].

One of these patient groups is Schizotypal Personality Disorder (SPD), which is characterized by pervasive deficits in social relatedness and communication, odd, magical, or paranoid thinking, distortions in perception, eccentricities, changes in affect, and social anxiety [10, 11]. Schizotypal Personality Disorder is thought to typically emerge in late adolescence and the prevalence in childhood is yet to be specifically investigated [10, 12]. Despite this, there is preliminary evidence that SPD symptoms can emerge between 6 and 12 years of age [8, 13–15], which has led to increased attention on this cohort. These symptoms are similar to the schizophrenia prodrome and the “at risk mental state” for psychosis [16]. Also, adults and adolescents with SPD have an increased risk of developing psychosis [17, 18]. Investigating SPD symptoms during childhood may provide a valuable avenue to better understand the development of SSDs. Indeed, preliminary longitudinal evidence in two independent studies suggests that children with SPD have a 17–25% chance of developing psychosis within a one- to three-year period [8, 19].

The clinical presentation of SPD in childhood is not well understood. Literature has described features including social interaction deficits, solitary tendencies, odd speech and ideation, formal thought disorder, unusual perceptions, magical thinking, and preoccupations with bizarre fantasies and interests [8, 14, 15, 19–22]. Whilst these symptoms are aligned with the adult DSM-IV-TR and DSM-5 criteria (note that criteria for SPD are relatively unchanged since the third edition of the DSM) [10, 12, 23], additional clinical features emerge during childhood including motor delays and difficulties maintaining and shifting attention [14, 15, 20]. Furthermore, the diagnostic criteria for SPD are yet to be operationalized for use in childhood [14]. To date, criteria surrounding social, interpersonal, and language symptoms of SPD have only partially been defined in childhood [14, 15, 21, 22]. Additional research is required to more thoroughly characterize these symptoms in childhood and their long-term outcome. Thus, (1) identifying and characterizing these symptoms in children will assess the prevalence of these symptoms and disorder in childhood; (2) longitudinal studies will be important to understand how these features in childhood evolve into adolescence, including the emergence of psychosis or other outcomes, including functional impairment; (3) interventions to improve functioning in a group of children with marked functional disability will benefit from work to better characterize this disorder.

In addition, the criterion of bizarre fantasies is not specific and there is little information in diagnostic manuals about how this might present in children [10, 12]. Some research has shown that imaginary companions and a proneness to fantasize are related to increased schizotypal symptoms [24, 25]. In support of this, Testa et al. [26] described make-believe worlds, invisible friends, a relationship with inanimate objects, and visions of imaginary monsters in children with learning difficulties that may fit DSM-IV-TR criteria for SPD. Other characteristics, such as odd or eccentric behavior, ideas of reference, and unusual perceptual experiences, are also overly general in their descriptors and need to be better defined into clinically identifiable behaviors in children aged between 6 and 12 years.

To date, the majority of childhood investigations have applied the DSM criteria to identify children with SPD. Two longitudinal studies have demonstrated moderate diagnostic stability across one-, two-, and three-year follow-up periods [8, 19]. This level of stability is encouraging but is likely to be affected by the limited operationalization of diagnostic criteria for children. Thus, additional investigation of the clinical phenotype of SPD is necessary to continue this work.

A second approach used to identify children with schizotypal traits is to adapt adult measures of schizotypy for use with children: Schizotypal Personality Questionnaire-Child (SPQ-C) [27] and Schizotypy Traits Questionnaire-Child (STA-C) [28]. The SPQ-C is aligned with DSM-IV criteria for SPD and comprises three factors: cognitive/perceptual difficulties, interpersonal difficulties, and disorganized symptoms [27]. In contrast, the STA-C assesses the positive symptoms of schizotypy: unusual perceptual experiences, paranoid ideation/social anxiety, and magical thinking [28]. The factor structures of both scales are comparable to the adult measures but have not been investigated in childhood clinical samples, which may demonstrate a different result [29]. Further, the adapted scales are based upon adult personality disorders and do not include observational data and informant reports, which are recommended for assessing childhood personality traits [30].

To overcome these methodological issues, the current authors developed the Melbourne Assessment of Schizotypy in Kids (MASK), a semistructured assessment designed to capture the clinical features of childhood SPD. This tool gathers information obtained from three sources to describe evident symptomology: the parent, the child, and observations by the treating clinician. This is similar to other pediatric assessment instruments (Gardner et al. [31]). Items on the MASK are largely based on DSM-IV criteria but also include features of childhood SPD that have been described elsewhere including motor deficits and bizarre fantasies [14, 15, 20].

In developing this measure, it was important to consider neurodevelopmental disorders that present with similar symptomology including Autism Spectrum Disorder (ASD). Wolff and Barlow [20] undertook the only known direct comparison of childhood SPD and ASD. They found great overlap in symptoms; however, only the SPD group was fixated on fantasies and imaginations [20]. Adults with SPD also present with a history of autistic traits during childhood [32], whilst adolescents with ASD record higher scores than controls on the SPQ [33].

The main objectives of the present study were to investigate the psychometric properties of the MASK in three samples: SPD, ASD, and typically developing (TD) children, and use this measure to describe the clinical phenotype of SPD in childhood. It was predicted that the MASK would provide a consistent measure of schizotypal symptoms across all groups; that children with SPD would obtain the highest MASK scores, followed by ASD and then TD children; that the MASK total score would accurately differentiate between children with SPD and those with ASD; and that the MASK would have a similar three-factor structure to the DSM-IV aligned SPQ-C. It was also expected that childhood SPD would be defined by similar characteristics to SPD in adults, with the addition of unusual fantasies, motor delays, and attention difficulties.

## 2. Method

### 2.1. Participants

Sixty-eight children aged between 5 and 12 years (*M* = 9.66, SD = 2.12) were recruited from pediatric services and the general community in Melbourne. Children were allocated to one of three groups: SPD (*n* = 21), ASD (*n* = 15), and TD (*n* = 32), and were excluded if they had a previous head injury (defined as a loss of consciousness greater than 5 minutes) or a known neurological condition (Table 1).

Subjects were allocated to the SPD group if they met DSM-IV-TR criteria for SPD (diagnosed by one of the authors; Tonge: child psychiatrist, or Testa: neuropsychologist) [10]. To establish diagnostic reliability, authors, Tonge and Testa, independently diagnosed eight children in this group (38%) and compared diagnoses. Of those with SPD, 13 children had previously received a diagnosis of ASD, two a diagnosis of Attention Deficit Hyperactivity Disorder (ADHD), and one a diagnosis of Reactive Attachment Disorder. Exclusion based on these preexisting diagnoses was not undertaken due to this high comorbidity rate within the SPD group. Nine children in the SPD group were taking one or more psychotropic medications (8× risperidone, 3× fluoxetine, and 1× imipramine), and two were taking sodium valproate. Those in the ASD group had previously received a DSM-IV-TR criteria diagnosis of either Asperger's Disorder (*n* = 8) or Autistic Disorder (high functioning, i.e., intellectual ability formally assessed to be in the normal range) (*n* = 7) from a multidisciplinary Autism assessment clinic. None of the controls had previously received a diagnosis of a psychiatric disorder, learning delay, or developmental delay. Written informed consent was obtained from at least one parent or guardian of each child. The project was approved by Melbourne Health Research Ethics Committee and Monash University Research Ethics Committee.

Children were administered the Wechsler Preschool and Primary Scale of Intelligence (WPPSI-III; less than 6 years of age) or the Wechsler Intelligence Scale for Children-Fourth Edition (WISC-IV; children 6 years and over) to estimate intelligence (Table 1: data not available for two of the SPD participants) [34, 35]. Because approximately 40% of participants had a 10-point or greater discrepancy between their verbal and nonverbal abilities, full scale IQ scores were considered invalid [35].

### 2.2. Measures

#### 2.2.1. The Melbourne Assessment of Schizotypy in Kids (MASK)

The MASK is a semistructured tool that was developed to measure SPD features in children aged between 5 and 12 years. The MASK comprises three components: Background Interview, Child Clinical Interview, and Clinical Presentation Checklist. The Background Interview is conducted with the parent or guardian of the child and documents the child's developmental history regarding schizotypal symptoms. The introductory interview from the Schedule for Affective Disorders and Schizophrenia for School-Age Children-Present and Lifetime Version (K-SADS-PL) [36] was also used in conjunction with the Background Interview of the MASK to provide a more general developmental history.

The Child Interview consists of questions that explore features of childhood SPD. These were developed from various sources including adult and child measures of schizotypy [28], psychiatric interviews for children [37], the K-SADS-PL [36], and previous research on fantasy proneness [38] and imaginary companions [39]. The latter components of fantasy were included to capture bizarre fantasies that have been reported in SPD but are not well defined. The Child Interview follows a semistructured format that is designed to provide a structure and framework without restricting the exploration of bizarre ideas, off-topic references, and additional symptomology. This exploratory approach was deemed necessary for children who may have difficulty understanding complex concepts or be guarded about their thoughts.

The Clinical Presentation Checklist includes 57 observable features of childhood SPD that are assembled within nine domains: social anxiety (items 1–6); social skills (items 7–12); motor abilities (items 13–17); language/though/ideation (items 18–27); fantasy/magical thinking (items 28–38); unusual perceptual experiences (items 39–42); behaviour (items 43–46); attention (items 47–52); and affect (items 53–57). Unequal items in each group were unavoidable due to the varying presentation of some symptomology and limited research on other characteristics. Each item is rated on a Likert scale (Never, Sometimes, Often, and Always) by the child's clinician only after obtaining information from both the child and the parent. The minimum score on the MASK is 57 and the maximum is 228. It takes approximately 1 hour and 30 minutes to administer the MASK in full.

#### 2.2.2. Behavior Rating Scales

The Behavioural Assessment System for Children-Second Edition (BASC-II) [40] and Conner's Rating Scale-Revised (CRS-R) [41] were also administered. The BASC-II is a parent and teacher questionnaire that appraises behavior and mood in children: Externalizing Problems; Internalizing Problems; Behavioral Symptoms Index; Adaptive Skills; and School Problems. The CRS-R assesses DSM-IV-TR symptomology of ADHD. The current study used three indices from both the parent and teacher forms: DSM-IV: Inattentive Type; DSM-IV: Hyperactive-Impulsive Type; and DSM-IV: Total [41].

### 2.3. Procedure

Sixty-six family groups (parent and child) were administered the MASK and introductory interview from the K-SADS-PL. Each child was also administered either the WISC-IV or WPPSI-III depending on their age. As part of a broader research project, sixty-one of these children were administered a battery of neuropsychological tests (Jones [42]). The remaining two participants were rated retrospectively using data from neuropsychological reports and clinical information provided by their treating clinician. The examiner then completed the MASK checklist. Child interviews were video recorded so that a second rater could recode 15 (22%) participants on the MASK. This subset included seven with SPD and four each with ASD or deemed TD. Detailed notes from parent interviews were also provided to this second rater. The second rater was blind to the diagnosis of six (9%) of the recoded children due to their involvement in recruitment and was not privy to any child's original score.

After each assessment, parents and teachers were asked to complete the BASC-II and CRS-R. Between 57 and 68 percent of forms were available for analysis.

### 2.4. Statistical Analysis

Data were analyzed using the IBM Statistics Package for the Social Sciences (SPSS, version 19). Cronbach alpha coefficients were calculated for the total MASK and its nine subscales to examine internal consistency. Intraclass correlation coefficients with two-way mixed models were used to determine the absolute agreement between two raters for 15 participants overall and 6 participants whose diagnosis was unknown to the second rater. Correlation coefficients between the MASK total score and raw subscale scores from the BASC-II and CRS-R were calculated to assess convergent validity.

An exploratory factor analysis of the MASK was undertaken using principal axis extraction with an oblique rotation (promax). Three a priori parameters were set to help maximize this analysis given the relatively small sample: loadings were limited to 0.4 and above; the smallest factor solution was preferred; and all MASK items were retained in the final analysis [43]. Scrutiny of the correlation matrix revealed that the majority of coefficients between items were 0.3 or greater. The Kaiser-Meyer-Olkin measure of sampling adequacy was 0.66 and Bartlett's test of sphericity was statistically significant (*χ* ^2^ = 5045.88, df = 1596, *P* < 0.001), both of which support the use of factor analysis [44].

Regressions were used to test for group differences, except in the case of gender, which was subjected to a chi-square analysis. Age and measures of intellectual abilities (visual, verbal, processing speed, and working memory) were assessed for skewness and kurtosis and considered appropriate for standard multiple regression analyses (i.e., values fell between 1 and −1). No data points fell ±3.29 SD's from the mean [44]. Conversely, MASK scores represent a count that is more appropriately analyzed using either a negative binomial regression when the variance is greater than the mean or a Poisson regression when the mean and variance are approximately equal [31]. A negative binomial regression was used to test MASK total scores and the majority of MASK subscales. The unusual perceptual experiences and affect subscales were subjected to a Poisson regression. These analyses included visual intellectual skills, verbal intellectual skills, processing speed, and working memory as covariates. To control for heteroscedasticity in the data, robust standard errors were adopted. Group differences on each factor were calculated using multiple regression with visual intellectual skills, verbal intellectual skills, processing speed, and working memory as covariates. Alpha levels were kept at 0.05 because the analyses were considered exploratory.

A receiver operating characteristic (ROC) curve was computed to further investigate the MASK's ability to discriminate between children with SPD and those with ASD. For this analysis, the TD group was not included to more accurately reflect the clinical context within which this tool is intended for use. A ROC curve plots the true positives against the false positives in order to determine the capacity of a test to accurately predict a child's diagnostic category. The area under the curve (AUC) was examined. This analysis was also used to reveal possible cut-off scores for the MASK. The sensitivity and specificity of each cut-off score were calculated. Sensitivity refers to the percentage of children with a diagnosis of SPD that were correctly identified using their MASK total score, whereas specificity refers to the percentage of children that did not meet criteria for SPD that were correctly identified as not having SPD.

## 3. Results

### 3.1. Internal Consistency

The Cronbach alpha coefficient for the 57 MASK items was 0.98. Alpha coefficients for the nine subscales of the MASK were social anxiety (0.86); social skills (0.94); motor abilities (0.92); language/thought/ideation (0.93); fantasy/magical thinking (0.86); unusual perceptual experiences (0.85); behaviour (0.88); attention (0.93); and affect (0.73).

### 3.2. Interrater Reliability

The intraclass correlation coefficient between two MASK raters for 15 participants was 0.980 (95% CI: 0.941–0.993, *P* < 0.001), indicating 98% agreement between raters. The intraclass correlation coefficient for 6 participants was similar and also significant (0.990 (95% CI: 0.938–0.999, *P* < 0.001)).

### 3.3. Group Differences on the MASK

**Figure 1:**
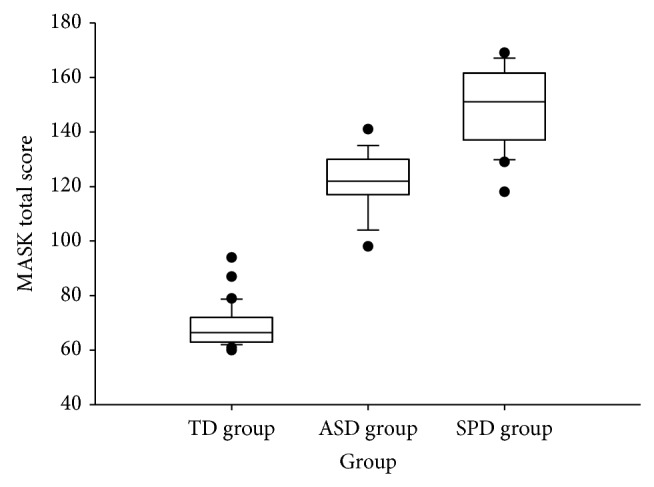
A comparison of the distribution of scores on the Melbourne Assessment of Schizotypy in Kids (MASK) for each group. Note: boxes represent the interquartile range, whiskers represent 1.5x the interquartile range, and the bisecting line in each box represents the median. Black dots represent outlying data points. ASD = Autism Spectrum Disorder, SPD = Childhood Schizotypal Personality Disorder, and TD = typically developing.

Independent of verbal intellectual skills, visual intellectual skills, processing speed, and working memory (*β* = −0.003, Wald *χ* ^2^(58) = 6.22, *P* < 0.02), MASK scores for children with SPD were significantly higher than for the TD and ASD groups (Figure 1 and Table 2). MASK scores for the ASD group were significantly greater than TD children.

The SPD group recorded higher scores than the TD group on each MASK subscale (Table 2). Children with SPD also recorded greater scores than those with ASD on the language/thought/ideation, fantasy/magical thinking, unusual perceptual experiences, and behaviour and affect subscales. The ASD group recorded greater scores than the TD group on each subscale except fantasy/magical thinking and unusual perceptual experiences. To summarize the significant covariate results for subscale analyses, working memory difficulties were associated with lower scores on the social skills subscale (*β* = −0.005, Wald *χ* ^2^(58) = 6.94, *P* < 0.009) and on the fantasy/magical thinking subscale (*β* = −0.004, Wald *χ* ^2^(58) = 4.89, *P* < 0.03).

### 3.4. MASK Factors

An initial principal axis analysis (eigenvalues greater than 1) revealed 11 factors that explained 82.83% of the variance. A subsequent parallel analysis (using permutations of the raw data) revealed that a two-factor solution was optimal, which explained 54.12% of the variance (Table 3). Items loading on the first factor, which explained 43.93% of the variance, represent social/pragmatic symptoms (social skill deficits, social anxiety, pragmatic language difficulties, and attention deficits). Items loading on the second factor accounted for 10.18% of the variance and represented positive schizotypal symptoms (disorganized/paranoid/magical thinking, bizarre fantasies, and unusual perceptual experiences). Five items did not load on either factor (Table 3), which may be indicative of item redundancy.

Both the SPD (*β* = 1.70, *t*(58) = 12.94, *P* < 0.001) and ASD (*β* = 1.80, *t*(58) = 12.10, *P* < 0.001) groups scored significantly higher than TD controls on the social/pragmatic symptoms factor. There was no difference between ASD and SPD. On the positive schizotypal symptoms factor, the SPD group scored significantly higher than TD (*β* = 1.86, *t*(58) = 10.66, *P* < 0.001) and ASD (*β* = 1.74, *t*(58) = 10.02, *P* < 0.001). None of the covariates were significant predictors of either MASK factor.

### 3.5. Validity Testing

Moderate to high correlations were observed between the MASK and BASC-II/CRS-R subscales (Table 4), indicating satisfactory convergent validity.

The area under the curve in the ROC analysis was 0.98 (*P* < 0.001; 95% CI: 0.86–1.00), which suggests that the total MASK score was an excellent gauge of SPD.

Three potential cut-off scores for the MASK are presented in Table 5. This table also displays the sensitivity and specificity values for each of these cut-off scores and the number of children scoring below and above these cut-offs. The results indicate that a cut-off of 132 maximizes both the sensitivity and specificity of the MASK for children with SPD or ASD.

## 4. Discussion

The main objectives of this study were to investigate the reliability of a newly developed assessment tool for childhood SPD (the MASK) and use this measure to characterize the clinical phenotype of childhood SPD. The occurrence of SPD in childhood represents an important opportunity to better understand the developmental trajectory of SSDs. We combined DSM-IV-TR criteria for SPD and previous research on childhood SPD to develop a semistructured assessment that is suitable for use with children.

Total scores on the MASK are internally consistent and convergent validity was adequate when compared to other measures, although both of these findings require replication. In support of discriminant validity, children with SPD recorded significantly higher MASK scores than both the ASD and TD groups, and children with ASD scored significantly higher than controls. This was expected, given that SPD DSM-IV criteria include ASD symptomology [10]. It is also encouraging that scale items designed to capture aspects of SPD that are either reported elsewhere or that lack operationalization in the DSM-IV were also consistent and reliable in the current sample. Of most interest was the predominant feature of bizarre fantasies. The internal consistency of bizarre fantasy items was good suggesting that they accurately captured these features. The MASK also had good construct validity, particularly with regard to its use in clinical populations. Two main factors were identified comprising social/pragmatic symptoms and positive schizotypal symptoms. This is inconsistent with previous research on schizotypy in children, where a three-factor solution is described: cognitive/perceptual difficulties; interpersonal difficulties; and disorganized symptoms [27]. It is apparent that MASK items that might otherwise be associated with a “disorganized symptoms” dimension have loaded on the first and second factor. This might be attributable to the clinical samples examined, rather than the normative samples used in previous investigations [27]. Adult investigations suggest that a three-factor model of schizotypal symptoms may not be directly applicable to clinical groups [29].

Children with SPD also presented with characteristics that distinguished them from the autism spectrum. Children with either SPD or ASD had more social/pragmatic symptoms compared to controls, while the SPD group presented with more positive schizotypal symptoms than children with ASD and controls. This is an important finding that highlights distinct differences between these developmental disorders. Barneveld et al. [33] also showed that negative/interpersonal schizotypal symptoms are more common in adolescents with ASD than positive schizotypal symptoms. These similarities and differences may help to explain why only two factors underlie the MASK in the current study. Further research is required to replicate this two-factor solution in a larger sample, especially given the limitations of factor analyses in small samples.

Additionally, the current analysis of two clinical samples suggests that the total score on the MASK is an excellent indicator of a DSM-IV-TR diagnosis of SPD. Given that there are limited differences between DSM-IV-TR and DSM-5 criteria for SPD, the MASK is also applicable to DSM-5 diagnoses. The results also indicate that a cut-off score of 132 maximized diagnostic sensitivity (90.48%) and specificity (93.33%). Given these promising preliminary findings, it will be important for future research to explore the clinical efficacy of the MASK as a diagnostic tool in a community-based clinical sample that includes children with a range of diagnoses (e.g., child onset schizophrenia, ADHD, depression, and anxiety).

With regard to the final objective of this paper, the pattern of results on the MASK subscales helps to better understand the clinical phenotype of childhood SPD. The SPD group showed heightened social anxiety, poor social and pragmatic language skills, motor delays, disorganized, tangential, paranoid, and magical thinking, preoccupations with fantasies, unusual perceptual experiences (auditory, visual, and olfactory), eccentric behavior, difficulties maintaining and shifting their attention, and changes in affect compared to TD children. While this is consistent with previous characterizations of childhood SPD [8, 14, 15, 19–22], to the authors knowledge, this is the first study to operationalize the DSM-IV criteria for childhood SPD in full. Future research should monitor children with SPD across development to better understand developmental changes in symptoms within a longitudinal investigation. This would permit an in-depth examination of the symptomology of bizarre fantasies given evidence suggesting that imaginary companions and related fantasies have a beneficial impact on development in TD children [39, 45]. It is possible that the level of preoccupation in childhood SPD coupled with a tendency to experience unusual perceptions may contribute to preoccupations with fantasies. In addition, their inability to shift from and inhibit these thoughts and ideas points to underlying cognitive deficiencies.

Two methodological issues are apparent. First, more than half of the SPD group had previously received a diagnosis of ASD. This may have resulted in more similarities between the experimental groups than what might ordinarily be present. The presence of other comorbidities such as ADHD might also compound the findings. Second, nine children in the SPD group were taking psychotropic medication at the time of their assessment. Adult findings indicate that atypical antipsychotic medications (such as risperidone) reduce both positive and negative symptoms of SPD [46]. Both of these limitations may have reduced the variance on the MASK between ASD and SPD groups. Further work is needed to control for these limitations, though this may be difficult given the complex clinical nature of children with such disorders.

## 5. Conclusion

The current analysis suggests that childhood SPD is characterized by a complex set of symptoms that makes it difficult to identify in young children, particularly when distinguishing it from ASD. Nevertheless, the current results provide a detailed operationalization of DSM-IV-TR criteria for SPD in childhood. By extension, limited changes to SPD criteria in DSM-5 suggest that the MASK is also applicable to DSM-5 use. The MASK is designed to assist health professionals in exploring these complex characteristics in a developmentally appropriate way. To the authors' knowledge, the MASK is the first structured and standardized clinical assessment for SPD that is suitable for children aged between 5 and 12 years and incorporates a number of characteristics that have been associated with childhood SPD but are not included in diagnostic manuals.

## Supplementary Material

Refer to supplementary material for a copy of the Melbourne Assessment of Schizotypy in Kids (MASK).

## Figures and Tables

**Table 1:** Descriptive statistics for age and intellectual skills for each experimental group.

Variable	SPD group	ASD group	TD group	Between group differences
% Female	38%	33%	46.8%	Nil
	M (SD)	M (SD)	M (SD)	
Age (years)	9.79 (2.52) *n* = 21	8.93 (2.05) *n* = 15	9.93 (1.83) *n* = 32	Nil
Verbal intellectual skills	97.37 (15.50) *n* = 19	93.80 (17.37) *n* = 15	109.81 (12.73) *n* = 32	TD > SPD^**^, ASD^**^
Visual intellectual skills	99.37 (12.28) *n* = 19	98.60 (10.31) *n* = 15	107.31 (11.81) *n* = 32	TD > SPD^*^, ASD^*^
Processing speed	93.11 (13.34) *n* = 19	91.53 (9.82) *n* = 15	108.31 (12.89) *n* = 32	TD > SPD^***^, ASD^***^
Working memory	95.28 (14.04) *n* = 18	90.13 (11.57) *n* = 15	112.41 (12.43) *n* = 32	TD > SPD^***^, ASD^***^

*Note.* ^*^ *P* < 0.025, ^**^ *P* < 0.01, and ^***^ *P* < 0.001. Working memory was not measured in children less than 6 years of age. ASD: Autism Spectrum Disorder; SPD: Schizotypal Personality Disorder; TD: typically developing.

**Table 2:** Descriptive statistics for the Melbourne Assessment of Schizotypy (MASK) total score and subscale scores in each group.

	Group (*n*)					
	TD (32)		ASD (15)		SPD (21)	
	M (SD)	Median	M (SD)	Median	M (SD)	Median
MASK total^a,b,c^	68.81 (7.70)	66.50	121.00 (10.45)	122.00	149.95 (14.23)	151.00

MASK subscale						
Social anxiety^a,b^	8.03 (2.21)	7.00	15.93 (3.24)	16.00	15.90 (3.00)	16.00
Social skills^a,b^	7.13 (1.29)	7.00	19.07 (2.09)	20.00	18.67 (2.18)	19.00
Motor abilities^a,b^	5.50 (1.12)	5.00	13.80 (2.24)	14.00	12.29 (4.57)	13.00
Language/thought/ideation^a,b,c^	10.72 (1.20)	10.00	15.47 (3.04)	15.00	26.71 (4.48)	28.00
Fantasy/magical thinking^a,c^	15.09 (2.64)	14.00	18.27 (3.28)	10.78	26.71 (5.87)	28.00
Unusual perceptual experiences^a,c^	5.22 (0.94)	5.00	4.60 (0.63)	5.00	9.81 (3.79)	10.00
Behaviour^a,b,c^	4.44 (0.98)	4.00	9.00 (1.81)	9.00	12.62 (1.77)	13.00
Attention^a,b^	7.34 (1.96)	6.00	17.13 (3.27)	18.00	16.43 (4.27)	16.00
Affect^a,b,c^	5.41 (0.76)	5.00	7.73 (1.75)	7.00	10.81 (2.02)	11.00

*Note.* ^a^ *P* < 0.001 for comparison between SPD and TD groups; ^b^ *P* < .001 for comparison between ASD and TD groups; and ^c^ *P* < .001 for comparison between SPD and ASD groups. All analyses were conducted using either a Poisson or negative binomial regression model with verbal intellectual skills, visual intellectual skills, processing speed, and working memory as covariates. ASD: Autism Spectrum Disorder; M: mean; SD: standard deviation; SPD: Schizotypal Personality Disorder; TD: typically developing.

**Table 3:** Obliquely rotated factor loadings on two schizotypal factors extracted from the Melbourne Assessment of Schizotypy in Kids (MASK).

MASK item	Factor 1	Factor 2
Has/displays difficulties completing fine motor tasks (e.g., has trouble writing neatly)	0.993	
Has/displays difficulties when fine motor skills are required (e.g., manipulating, buttons, tools, and utensils)	0.934	
Avoids eye contact during first session with clinician	0.926	
Bases conversation topics on own interests	0.902	
Has difficulty switching from own interests to other topics or activities	0.897	
Bases play themes on own interests	0.867	
Fails to demonstrate the reciprocal nature of conversation (e.g., does not take turns)	0.867	
Is clumsy while completing tasks	0.860	
Finds it difficult to communicate and socialise with other kids	0.847	
Has/displays difficulties learning new motor skills after repeated attempts	0.807	
Presents with difficulties self-directing focus of attention to salient information	0.796	
Is fidgety or restless	0.791	
Has difficulties doing gross motor tasks like riding a bike or playing sport	0.786	
Shows difficulty remaining focused on activities	0.777	
Experiences/displays feelings of unease or discomfort when meeting new people	0.771	
Feels/displays discomfort in situations where there are a lot of people around	0.730	
Has difficulty shifting from one focus of attention to another focus (e.g., difficulty giving two part answers on comprehension)	0.710	
Has difficulties sitting still or remaining seated	0.698	
Has difficulty attending to conversations	0.690	
Sticks to themselves in group situations (e.g., parties)	0.667	
Has few close friends that are not members of their immediate family	0.665	
Daydreaming distracts them from completing tasks	0.628	
Prefers to play alone rather than with friends	0.628	
Is disorganised when undertaking tasks	0.595	
Speech contains odd uses of intonation, rhythm, and stress, or these aspects are absent	0.581	
Has little difficulty entertaining themselves while alone	0.479	
Seems overly excited to share information	0.439	
Appears inappropriately happy or elated	0.422	
Speech is either overly concrete or overly abstract	0.411	
Interprets innocuous or irrelevant events as being personally salient		0.942
Is paranoid or suspicious about innocuous or irrelevant events		0.929
Is preoccupied with these fantasies to the point where behaviour is influenced		0.910
Reports hearing voices/sounds that are not based on reality		0.894
Describes a make-believe world or place as if it were real		0.867
Has paranoid or suspicious ideas about the behaviour and motives of others		0.861
Expresses odd or bizarre ideas in speech		0.838
Imaginary characters, creatures, or events appear important to the child, more so than actual friends or events		0.833
Is described by others as being peculiar or eccentric		0.797
Refers to imaginary characters, creatures, or events		0.776
Refers to a make-believe world or place		0.764
Reports enhanced, altered, or perplexing hearing, sight, smell, or touch		0.700
Displays signs of culturally odd or bizarre behaviour in social settings		0.681
Shows difficulties sticking to one topic within sentences		0.666
Loses track of what they are saying		0.657
Appears resentful, irritable, or angry		0.596
Reports seeing images/visions that are not based on reality		0.589
Speech content is elaborated out of context, when others are no longer engaged in conversation		0.580
Reports sensing smells or tactile sensations that are not based on reality		0.545
Experiences vivid daydreams		0.527
Content of speech deviates from original topic (tangential)		0.515
Demonstrates incongruous or inappropriate facial expressions		0.482
Believes they have super- or magical-powers		0.448
Appears depressed, dejected, or downcast		0.448
Describes mythical/cartoon characters depicted in stories and movies as if they are real		0.413
Appears guarded and is reluctant to share personal information		
Believes they have a sixth sense		
Shows restricted range of facial expressions when engaging in a conversation		

*Note.* Factor 1: social/pragmatic symptoms; Factor 2: positive schizotypal symptoms.

**Table 4:** Pearson correlation coefficients between parent and teacher rating scales and the Melbourne Assessment of Schizotypy in Kids (MASK).

Scale	N	MASK total score (r)
BASC-II—parent scales		
Externalising problems	46	.54^***^
Internalising problems	46	.53^***^
Behavioral symptoms index	46	.81^***^
Adaptive skills	46	−.72^***^
BASC-II—teacher scales		
Externalising problems	43	.58^***^
Internalising problems	43	.49^**^
School problems	43	.60^***^
Behavioral symptoms index	43	.72^***^
Adaptive skills	42	−.66^***^
CRS-R—parent scales		
DSM-IV inattentive	39	.76^***^
DSM-IV hyperactivity	39	.65^***^
DSM-IV ADHD total	39	.77^***^
CRS-R—teacher scales		
DSM-IV inattentive	43	.57^***^
DSM-IV hyperactivity	43	.48^**^
DSM-IV ADHD total	43	.56^***^

*Note.* ^**^ *P* < .01; ^***^ *P* < .001. ADHD: Attention Deficit Hyperactivity Disorder; BASC-II: Behavioral Assessment Scale for Children-Second Edition; CRS-R: Conner's Rating Scale, Revised; DSM-IV: Diagnostic and Statistical Manual of Mental Disorders-Fourth Edition.

**Table 5:** Sensitivity and specificity data for alternative cut-off scores for the Melbourne Assessment of Schizotypy in Kids (MASK) total score in the Schizotypal Personality Disorder (SPD) and the Autism Spectrum Disorder (ASD) groups only.

	MASK cut-off ≥ 127		MASK cut-off ≥ 132		MASK cut-off ≥ 143	
Sensitivity	95.24%		90.48%		71.43%	
Specificity	73.33%		93.33%		100.00%	

Actual diagnosis	Predicted diagnosis					
	SPD (*n*)	No SPD (*n*)	SPD (*n*)	No SPD (*n*)	SPD (*n*)	No SPD (*n*)

SPD	20	1	19	2	15	6
ASD	4	11	1	14	0	15
