# Supplementary material for: The Melbourne Assessment of Schizotypy in Kids: A Useful Measure of Childhood Schizotypal Personality Disorder
Source: Biomed Res Int. 2015 Jan 6;2015:635732. doi: 10.1155/2015/635732 (PMC4300034; doi:10.1155/2015/635732)
Supplement: Supplementary file 1 — Refer to supplementary material for a copy of the Melbourne Assessment of Schizotypy in Kids (MASK). [file 635732.f1.pdf]

The Melbourne Assessment of Schizotypy in Kids (MASK)

Family Background (parent interview)

The following questions are to be directed at the child's parent or guardian. These questions are designed to be used in conjunction with the standard background interview. This will assist completing the clinical presentation checklist.

Play Behaviour

- *What sort of games they like to play at home and at school?*
- *Does this involve make-believe play, or pretend play?*
    - *Is this make-believe based on movies/books or have they constructed it themselves?*
- *Can you describe these games?*
- *Do they play well on their own?*
- *Do they play with other children?*
- *Which do they prefer, alone or with others?*
- *When they play with other children, do they share their possessions?*
- *Do they play games with others only on their own terms (own rules, favourite games etc.)?*

Peer/Social Relations

- *Can you describe your child's behaviour at other children's parties?*
- *Have you noticed anything unusual about the way they interact with other children?*
- *Do they avoid social events like these?*
- *Do they get nervous, intimidated or overly shy when introduced to new people?*

Perception of others

- *How do teachers describe your child to you?*
- *Have they noticed anything unusual about your child's behaviour or commented that they are different from other children?*
- *Have people or teachers ever commented that your child is odd or eccentric?*

Imaginary Characters / Fantasy

- *Some children daydream more than others, does your child daydream?*
- *How often do they daydream?*
- *Do they seem distracted by their thoughts throughout the day or when they are playing alone?*
- *Do you know what they are thinking about when they daydream?*
- *Do you know if your child has an imaginary friend, imaginary characters or play with imaginary creatures?*
    - *How does your child feel about this imaginary character?*
    - *Can you describe their relationship?*
    - *How important are they to your child?*
- *Have they ever described an imaginary place or world to you?*
    - *Can you tell us about this?*
    - *Is this based on movies or books, or is it self-constructed?*

Child Interview

This semi-structured interview is suited for children aged between 5 and 12 years. The interview is broken into two parts. The first part involves a drawing task. The second part consists of a series of questions designed to explore different aspects of the child's thoughts and behaviours. This is to be used to assist the completion of the clinical presentation checklist.

Note that the following questions are a guide for conducting a clinical interview for schizotypal personality traits in children. It is not necessary to ask all the questions, nor is it necessary to ask them in a strict fashion.

It is important to explore the child's understanding of the questions. Odd answers can arise due to a lack of understanding. The groups of questions are designed to explore both the eccentricity of the answer and to ensure the child has understood the line of questioning.

**Take note of the child's speech and affect during the clinical interview.*

Drawing Exercise: Children are asked to draw a picture representing a sad/bad or happy dream (it is often appropriate to draw both dreams). They are then asked a series of questions about their drawings. The drawing task is adapted from Tonge, B. J. (1982). Draw a Dream: An intervention promoting change in families in conflict. In F. Kaslow (Ed.), *The International Book of Family Therapy* (pp. 212-225). New York: Brunner/Mazel.

*Take note of imaginary characters or creatures, the presence of a distinct fantasy world, detail/elaboration of fantasy world (if one present), bizarre ideation, their willingness to share this information, and their speech and affect during the interview.

"Now we are going to do a drawing activity where we both get a chance to draw something." The examiner begins to draw a simple bed with a child in it, with a large speech bubble emanating from the person in the bed. "*Here is a picture of a girl/boy named* ______ (insert the child's name) *in bed having a bad/sad or happy dream. Let us imagine that this is you. You draw me a picture of your sad/bad or happy dream here* (Gesturing toward the speech bubble)*."*

*Note that some children may find it difficult to think of a happy or sad/bad dream. In this situation, just get them to draw one or the other, or whatever they can think of that they've dreamt about.

Drawing Questions:

1. What's happening (gesture toward drawing) what is it about?
2. What is the best or worst part of the dream?
3. What happens next?
4. Is there a time in this dream where you would like to wake up?
5. How many times have you had this dream?
6. Where is this place (gesture to drawing)? What is it called?
7. Can you describe the world/place that you have drawn?
8. Is this only in your dreams?
9. How often do you play/go there?
10. How did you find this world/place? Did you create it?
11. How do you get to it?
12. How do you feel when you are there?
13. Do you ever daydream about this world/place?
14. When does this happen?
15. Do you find these daydreams distracting?
16. Can you draw this world/place for me?

If there are specific characters in the dream picture:

17. Who is this (point to a figure or in the case of symbol "What is this") or tell me about? How do you know them?
18. How often do you play with them? Every day or sometimes?
19. How do you feel when you play?
20. The following explore the child's relationship with the character in more depth.
    a. Do you play with _______ when you are not dreaming?
    b. What are ________ like, are they nice or mean, happy or sad, outgoing or shy etc.?
    c. Can you describe their family and friends?
    d. How long have you known ________, or how long have they visited you?
    e. Can you remember the first time you saw/met _______? What were you doing?
    f. What are some of the things you do with ________? Do you play with each other? What games do you play together?
    g. What toys do you play with?

21. *If the child does not draw an imaginary character or some other fictitious being in their dream ask them specifically*
    a. Do you have an imaginary friend or like to play with an invisible friend or animal?

22. *If the child refers specifically to an imaginary friend, and imaginary character, a mythical creature, or some other imaginary object or thing, explore their relationship with the following questions.*
    a. How do you meet/see them? Does ______ appear on their own or do you call them?
    b. What happens to _______ when other children are around?
    c. What do your friends at school think about ________, or are they a secret or invisible to your friends?

23. After this, you can explore specifics with the child about their fantasies or dreams. Using their language, discuss the fantasy aspects of these with the child to help ascertain degree of over-focus, paranoid beliefs, superstitiousness, magical thinking and affect.

Interview Questions

**Fantasy Proneness/Magical Thinking/Odd Beliefs**	
When you are playing with no-one else around, what sort of games do you play?<br>Can you tell me about some of the toys you like to play with?<br>Do your toys sometimes seem like they are alive?<br>What do you do if you have no toys to play with?	

Do you sometimes feel like you have nothing to do?<br>How does that make you feel?<br>Do you ever get bored?<br>When does this happen?<br>How often are you bored?<br>What do you do when you get bored?	

Sometimes kids find themselves daydreaming a lot, does this happen to you?<br>When does this happen the most? (at school, home etc.)<br>Does this distract you at school, home etc.?<br>What do you daydream about?<br>Can you describe these daydreams to me?<br>What are they like?<br>How clear are your daydreams?<br>Is it like watching a movie?	

What stories and movies have you read or seen recently?<br>What are your favourite stories?<br>Do you like fairy-tale creatures? Or superheroes?<br>Have you ever met one of these creatures or superheroes?<br>Can you tell me about this?<br>How did you feel when this happened?	

Some kids feel like they can talk to others with their mind.<br>Do you ever feel that other people know what you are thinking?<br>Or, have you ever felt that you can do this?<br>When has this happened to you?<br>Can you tell me how you do it?<br>What can this help you do?	

Unusual Perceptual Experiences	
Sometimes kids feel like they can smell, hear, taste and see more strongly than usual. Has this ever happened to you?<br>Can you tell me about this?<br>When did this happen to you?<br>Does it still happen?<br>How often does this happen?<br>What is it like?	

Do you remember thinking you heard or saw something that wasn't there?<br>What did you think made this happen?<br>Could it have been a trick or was it something else?<br>Has this happened at any other times?<br>When was this?	

**Social Anxiety**	
When you play games, who do you like to play with?<br>What sort of games do you play with your friends?<br>Can you tell me more about your friends?<br>How did you meet them?<br>When do you see them?<br>What sort of things do you do with your friends?<br>Do you talk to them a lot?<br>What sort of things do you talk about?<br>How do you feel when you are around your friends?	

Do you go to your friends' birthday parties?<br>What things do you do at these parties?<br>How do you feel when you are at these parties - happy, sad or bored?	

Do you meet new kids at parties?<br>What is it like meeting new kids?<br>What do you do when you meet new kids?<br>Do you get butterflies in your stomach?<br>What games do you play with kids you just met?<br>Do you find playing with them easy or hard?<br>Why do you think this is?<br>Is this the same for grown-ups you meet?<br>What is it like meeting them?	

Sometimes kids find it hard to talk to other kids.<br>Does this ever happen to you?<br>How much?<br>Can you tell me what happened?<br>What did you do when this happened?	

**Ideas of Reference/ Suspicious or Paranoid Ideation**	
When you see a group of kids talking, what do you do?<br>Do you ever think they are talking about you?<br>What makes you think this?<br>Has this happened before?<br>How many times has this happened?<br>What were they saying about you?	

Do you like people in your class?<br>Why/ Why not?<br>How do they feel about you?<br>What makes you think this?<br>Do you find yourself thinking about this a lot?<br>Do you worry about what others think about you?<br>Why/ Why not?	

Clinical Presentation Checklist

Clinical Observation

Integrate the information obtained from the Child Clinical Interview, the Background Interview and general observations made during the session to determine the extent to which their personality matches the characteristics listed below. Rate these behaviours using the following table and sum the item scores to establish the child's Total MASK Score.

Never	Sometimes	Often	Always
1	2	3	4

		1	2	3	4
	Social Anxiety				
1	Avoids eye contact during first session with clinician.				
2	Appears guarded and is reluctant to share personal information.				
3	Seems overly excited to share information.				
4	Experiences/displays feelings of unease or discomfort when meeting new people.				
5	Feels/displays discomfort in situations where there are a lot of people around.				
6	Sticks to themselves in group situations (e.g. parties).				
	Social Skills				
7	Fails to demonstrate the reciprocal nature of conversation (e.g. does not take turns).				
8	Bases conversation topics on own interests.				
9	Bases play themes on own interests.				
10	Prefers to play alone rather than with friends.				
11	Has few close friends that are not members of their immediate family.				
12	Finds it difficult to communicate and socialise with other kids.				
	Motor Abilities				
13	Is clumsy while completing tasks.				
14	Has difficulties doing gross motor tasks like riding a bike, or playing sport.				
15	Has/displays difficulties learning new motor skills after repeated attempts.				
16	Has/displays difficulties completing fine motor tasks (e.g. has trouble writing neatly).				
17	Has/displays difficulties when fine motor skills are required (e.g. manipulating, buttons, tools, utensils etc.).				
	Language/ Thought/ Ideation				

18	Shows difficulties sticking to one topic within sentences.				
19	Loses track of what they are saying.				
20	Content of speech deviates from original topic (tangential).				
21	Speech content is elaborated out of context or when others are no longer engaged in conversation.				
22	Speech contains odd uses of intonation, rhythm and stress, or these aspects are absent.				
23	Speech is either overly concrete or overly abstract.				
24	Expresses odd or bizarre ideas in speech.				
25	Interprets innocuous or irrelevant events as being personally salient.				
26	Has paranoid or suspicious ideas about the behaviour and motives of others.				
27	Is paranoid or suspicious about innocuous or irrelevant events.				
Fantasy/ Magical Thinking					
28	Refers to imaginary characters, creatures or events.				
29	Imaginary characters, creatures or events appear important to the child, more so than actual friends or events.				
30	Refers to a make-believe world or place.				
31	Describes a make-believe world or place as if it were real.				
32	Is pre-occupied with these fantasies to the point where behaviour is influenced.				
33	Have little difficulty entertaining themselves while alone.				
34	Daydreaming distracts them from completing tasks.				
35	Experiences vivid daydreams.				
36	Describe mythical/cartoon characters depicted in stories and movies as if they are real.				
37	Believe they have super- or magical-powers.				
38	Believe they have a sixth sense.				
Unusual Perceptual Experiences					
39	Reports enhanced, altered or perplexing hearing, sight, smell or touch.				
40	Reports hearing voices/sounds that are not based on reality.				
41	Reports seeing images/visions that are not based on reality.				
42	Reports sensing smells or tactile sensations that are not based on reality.				
Behaviour					
43	Is disorganised when undertaking tasks.				
44	Has difficulty switching from own interests to other topics or activities.				
45	Displays signs of culturally odd or bizarre behaviour in social settings.				

46	Is described by others as being peculiar or eccentric.				
	Attention				
47	Has difficulties sitting still or remaining seated.				
48	Shows difficulty remaining focussed on activities.				
49	Has difficulty attending to conversations.				
50	Is fidgety or restless.				
51	Has difficulty shifting from one focus of attention to another focus (e.g. difficulty giving two part answers on comprehension).				
52	Presents with difficulties self-directing focus of attention to salient information.				
	Affect				
53	Shows restricted range of facial expressions when engaging in a conversation.				
54	Demonstrates incongruous or inappropriate facial expressions.				
55	Appears depressed, dejected or downcast.				
56	Appears inappropriately happy or elated.				
57	Appears resentful, irritable or angry.				

Total MASK Score _____________
